# Interaction of Osteopontin with IL-18 in Obese Individuals: Implications for Insulin Resistance

**DOI:** 10.1371/journal.pone.0063944

**Published:** 2013-05-13

**Authors:** Rasheed Ahmad, Anfal Al-Mass, Dalal Al-Ghawas, Nada Shareif, Nadia Zghoul, Motasem Melhem, Amal Hasan, Fahad Al-Ghimlas, Said Dermime, Kazem Behbehani

**Affiliations:** 1 Immunology and Innovative Cell Therapy Unit, Dasman Diabetes Institute, Kuwait City, Kuwait; 2 Genetics and Genomics Unit, Dasman Diabetes Institute, Kuwait City, Kuwait; 3 Fitness and Rehabilitation Center, Dasman Diabetes Institute, Kuwait City, Kuwait; 4 Senior Management, Dasman Diabetes Institute, Kuwait City, Kuwait; Universidad Pablo de Olavide, Centro Andaluz de Biología del Desarrollo-CSIC, Spain

## Abstract

**Background/Objective:**

Osteopontin (OPN) and IL-18 are known inflammatory mediators and both participate in a wide range of biological processes linked to immunological disorders. Since an interaction between OPN and IL-18 has not been studied in obesity, we investigated whether: (i) their levels were simultaneously elevated in obese individuals; (ii) OPN was associated with IL-18 in obese individuals and (iii) their levels associated with fasting blood glucose (FBG) and BMI.

**Subjects and Methods:**

PBMCs and plasma samples were isolated from 60 individuals including lean as well as overweight and obese individuals. Subcutaneous adipose tissue samples were obtained. OPN and IL-18 were measured by ELISA. OPN and IL-18 mRNA expression was quantified by real time quantitative RT-PCR.

**Results:**

Obese individuals exhibited significantly increased circulating OPN levels as compared with lean individuals (obese 2865±101; lean 1681±116 pg/ml; P<0.0001). IL-18 levels were also high in obese individuals (obese 491±39, lean 301±26 pg/ml; P = 0.0009). OPN and IL-18 expression were simultaneously up-regulated (OPN: 5.4-Fold; IL-18: 8.9-Fold; P<0.05) in PBMCs from obese individuals compared to lean group. Adipose tissue from obese individuals had high expression of OPN (7.3-Fold) and IL-18 (9.6-Fold). Plasma OPN levels correlated positively with FBG levels (r = 0.32, P = 0.02). Similarly, IL-18 correlated positively with FBG levels (r = 0.406, P = 0.0042). Stepwise multiple regression analysis showed an independent association of BMI with OPN and IL-18. Interestingly, OPN levels increased progressively with an increase in IL-18 levels (r = 0.52, P = 0.0004). We also examined the regulatory role of IL-18 in OPN secretion from PBMCs. Neutralizing anti-IL-18Rα mAb reduced OPN secretion.

**Conclusion:**

These findings represent the first observation that plasma, PBMC and adipose tissue OPN and IL-18 are simultaneously increased and correlate with each other in overweight/obese individuals which may trigger the development of obesity-associated insulin resistance. Moreover, these results provide the direct evidence that IL-18 regulates OPN production in PBMCs.

## Introduction

Obesity induces chronic, low-grade tissue inflammation which is important in the development of obesity-related pathologies, such as insulin resistance and type 2 diabetes, and cardiovascular disease [1], [2], [3]. The cause and stimulus of persistent inflammatory activation in obesity remain largely unknown. Levels of circulating inflammatory biomarkers such as high-sensitivity C-reactive protein hsCRP, tumor necrosis factor receptor (TNF-αR), Interlukin-6 (IL-6), soluble tumor necrosis factor receptor 2 (sTNFr2), and cellular adhesion molecules are consistently elevated in the obese patients and normally correlate with parameters of adiposity, risk factors of cardiovascular diseases, insulin resistance, and type 2 diabetes risks [4], [5], [6], [7]. Adipocytes, monocytes, and macrophages are actively involved in the secretion of proinflammatory cytokines [8] 2010. In case of obesity, the levels of activated monocytes and macrophages are elevated. A positive correlation was found between body mass index (BMI) and percentage of macrophages, suggesting that fat tissue growth was associated with recruitment of blood monocytes. Activated monocytes infiltrate the adipose tissues and contribute significantly to sustain chronic inflammation through synthesis and increase of cytokines that may be etiologically intertwined with the mechanisms of obesity-induced insulin resistance [9], [10].

OPN is a multifunctional proinflammatory cytokine, a glycoprotein that is secreted by activated T cells, NK cells, dendritic cells and monocytes/macrophages and plays a major role in cell-mediated immunity [11], [12], [13]. OPN is one of the key factors which are actively involved in macrophage recruitment during inflammatory processes. It interacts with the integrin surface receptors through an Arg-Gly-Asp (RGD) sequence and with the CD44 receptor to induce signaling [14]. Macrophages at sites of inflammation are prolific producers of this cytokine which mediates monocyte adhesion, migration, differentiation, and phagocytosis [15], [16], [17], [18]. In adipose tissue, OPN induces infiltration and activation of macrophages and these infiltrated macrophages produce proinflammatory cytokines which contribute to adipose tissue insulin resistance [19].

IL-18 is a potent proinflammatory cytokine that belongs to the IL-1 superfamily and is produced by monocytes/macrophages and other cells [20]. In addition to monocyte/macrophages, IL-18 is also produced by adipose tissue [21] and higher circulating levels are found in obese than lean individuals. Both OPN and IL-18 participate in a wide range of potent inflammatory processes that are linked to immunological disorders. Mechanistically, IL-18 activates the transcriptional factors AP-1 and NF-κB in T lymphocytes and these factors are also involved in the transcriptional upregulation of OPN gene [22], [23], [24], [25]. Increasing evidence shows that circulatory levels of both inflammatory mediators are elevated in obese individuals [26], [27], [28], [29]. However, it is unclear whether both inflammatory mediators (cytokines) are simultaneously increased in obesity and also the interaction between two inflammatory mediators is so far not known. The data presented herein clarify the regulatory role of IL-18 regarding OPN expression in plasma, PBMC and adipose tissue, and also show their correlation with BMI and fasting blood glucose levels.

## Methods

### Participants and Clinical Parameters

Our department (Biomedical research) carried out a study with the collaboration of Fitness and Rehabilitation Center. Subjects were recruited by different advertisements. Participants were offered a cost free 6 month’s exercise program at Fitness and Rehabilitation Center. Before joining the program, subjects were examined at local clinic by medical physician. Subjects of both male and female were included if they (1) are older than 18 years with BMI less than 40 Kg/m^2^, (2) no previous exercise program at least one year prior to the study. Subjects were excluded if they (1) were morbid obese, (2) were unable to do exercise and (3) had major illness. Subjects who are diabetic were on antidibetic drugs (metformin, sulfourea, glitazone and exenatid. 60 adult individuals were divided into three groups including: (1) lean group: 16 individuals (2) overweight group: 20 individuals, and (3) obese group: 24 individuals. Written informed consent was obtained from all participants before enrolment in the study and the study protocol was approved by Ethical Review Committee of Dasman Diabetes Institute, Kuwait. Peripheral blood samples were collected for isolation of PBMCs and for determining blood glucose, total cholesterol, high density lipoprotein cholesterol (HDL-C), low-density lipoprotein cholesterol (LDL-C), and glycated hemoglobin A1C (HbA1C). Participants’ age, height, weight, blood pressure and diabetes status were also recorded at the time of blood collection. All biochemical tests were performed by using standard commercial kits. The participants’ characteristics are listed in table 1. Adipose tissue samples were also obtained from 9 (lean = 3, overweight = 3 and obese = 3) individuals with different BMI values.

**Table 1:** Characteristics of the study participants.

Characteristic	Lean (BMI<25)	Overweight (25<BMI<30)	Obese (30<BMI<40)
Age range (Years)	26–46	28–43	28–46
BMI	22.71±0.33	27.77±0.33	34.00±0.55
Glu (mmol/l)	4.91±0.17	6.12±0.47	5.84.±0.36
HbA1C (%)	5.48±0.08	6.34±0.44	6.05±0.2
Chol (mmol/l)	4.89±0.20	5.06±0.21	5.54±0.19
HDL (mmol/l)	1.24±0.08	1.2±0.07	1.14±0.06
LDL (mmol/l)	3.24±0.18	3.10±0.16	3.58±0.19
TGL (mmol/l)	0.82±0.13	0.80±0.17	1.79±0.20

### Collection of Serum/plasma and Peripheral Blood Mononuclear Cells (PBMCs)

Blood samples were collected from the individuals before they started exercise. For serum isolation, 5 ml blood was collected in vacutainer blood collection tubes and allowed to clot at room temperature for 30 minutes. Serum was obtained by centrifugation at 1000 rpm for 10 min (4°C). For PBMCs, blood was collected in EDTA tubes and mononuclear cells were isolated by using Ficoll-Hypaque density gradient centrifugation as described elsewhere [30]. Plasma was also collected, clarified by centrifugation, aliquoted and stored at −80°C until use.

### Subcutaneous Adipose Tissue Biopsy

Human adipose tissue was collected via abdominal subcutaneous fat pad biopsy lateral to the umbilicus using standard sterile technique. Adipose tissue samples were obtained from 9 individuals with different BMI. Tissue samples were stored in RNAlater RNA stabilization reagent (Qiagen) and stored at −80°C. Samples were then used for total RNA isolation.

### Stimulation of PBMCs and Cytokine Assay

PBMCs were cultured in RPMI 1640 medium (Invitrogen) supplemented with 10% or 2% FBS and antibiotics (streptomycin and penicillin). PBMCs were activated with rhIL-18 (R &D Systems/MBL) at a concentration of 20 ng/ml for 16 hours. Both supernatant and cells were collected for analysis of OPN expression.

### Neutralization of IL-18 Receptor with Antibody

PBMCs were incubated with IL-18Rα monoclonal antibody and isotype-matched control antibody (mouse IgG1; R&D Systems) at a concentration of 0.1 ug/ml for 30 minutes and then treated with rhIL-18 (20 ng/ml) for 16 hours. PBMCs and culture supernatant were analyzed for OPN mRNA and protein, respectively.

### Enzyme-linked Immunosorbent Assay

Concentrations OPN and IL-18 in plasma and culture supernatants were determined using commercial enzyme-linked immunosorbent assay kits (R & D Systems) according to the manufacturer's recommendation.

### Real Time Quantitative RT-PCR

Total RNA was extracted using RNeasy Mini Kit (Qiagen). The cDNA was synthesized using 1 ug of total RNA using high capacity cDNA reverse transcription kit (Invitrogen). 50 ng cDNA and 5 pmol of both forward and reverse primers were used in each real time PCR reaction. Real time PCR was performed using SYBR Green PCR Master Mix (Applied Biosystems) on 7500 real time PCR System (Applied Biosystems). All PCR reactions were performed in duplicate. The primers are used as follow: OPN Fwd (5′-AGGAGGAGGCAGAGCACA -3′) and OPN Rev (5′-CTGGTATGGCACAGGTGATG-3′); IL-18 Fwd (5′-GAAAATTTCAACTCTCTCCTGTG-3′) and IL-18 Rev (5′-CCTTCGTATGATGAAGATTCAAA-3′); GAPDH Fwd (5′-ATGGGGAAGGTGAAGGTC-3′) and GAPDH Rev (5′- GAGGTCAATGAAGGGGTCAT-3′). The mRNA levels were normalized against GAPDH mRNA and the amounts of OPN or IL-18 -mRNA relative to control were calculated with ΔΔCt-method [31]. Relative mRNA expression was expressed as fold expression over average of gene expression of lean group. The expression level in lean group was assumed to be 1. Values are presented as mean ± SEM.

### Statistical Analysis

Statsitical analysis was performed using GraphPad Prism software (La Jolla, CA, USA) and SPSS for Windows version 19.01 (IBM SPSS Inc., USA). Data are shown as mean ± standard deviation values, unless otherwise indicated. Unpaired Student t-test and Wilcoxon signed-rank test were used to compare means between groups. Correlation, linear regression and stepwise multiple regression analysis were performed to determine association between different variables. For all analyses, *P* value <0.05 was considered significant.

## Results

### Elevated Levels of Plasma IL-18 and OPN in Obese Individuals

**Figure 1:**
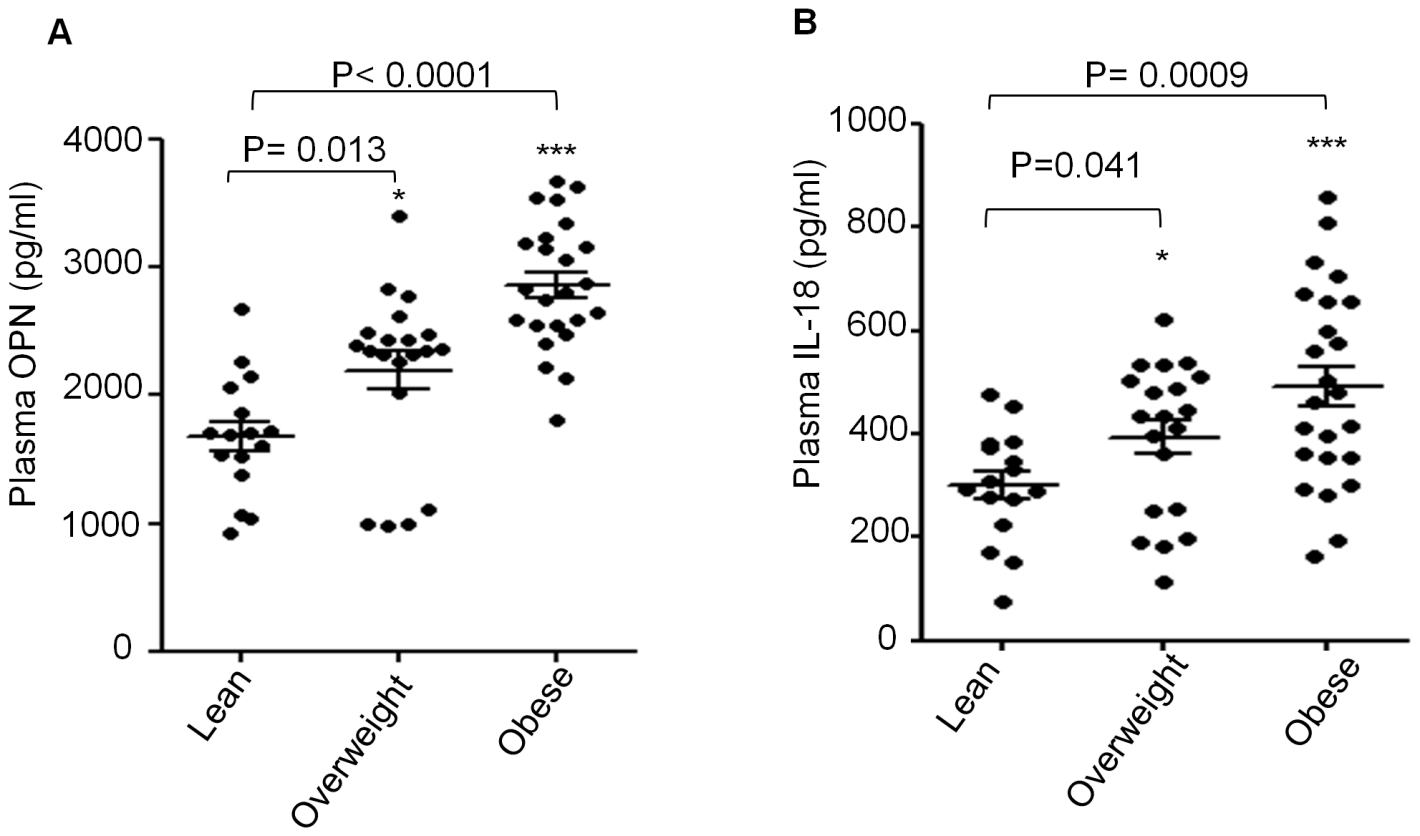
Plasma levels of OPN and IL-18 in obese individuals. 60 plasma samples from obese (n: 24), overweight (n: 20) and lean individuals (n: 16) were analyzed for expression of the circulating OPN (A) and IL-18 (B). Each dot represents the individual value of OPN and IL-18. Lines represented the mean values of plasma OPN and IL-18 of each group with ± SE.

IL-18 and OPN are considered important mediators of both immunological and pathological disorders. The levels of both mediators have been studied separately in obese individuals and have been found at high levels. Since no study has been done related to the measurement of these mediators in the same individuals, we conducted a cross-sectional study and measured simultaneously plasma levels of IL-18 and OPN from lean (n: 16), overweight (n: 20) and obese (n: 24) individuals. As shown in Fig. 1A and B, increased concentrations of OPN and IL-18 were found in obese as compared with lean individuals. Plasma from obese individuals showed significantly increased levels of OPN compared to lean individuals (obese 2865±101, overweight 2196±148, lean 1681±116 pg/ml; P<0.01). In parallel, plasma IL-18 levels were also increased (P = 0.0009) in obese individuals as compared with those of lean individuals (obese 491±39 versus 301±26 pg/ml, respectively).

### Increased Expression of OPN and IL-18 in PBMCs of Obese Individuals

**Figure 2:**
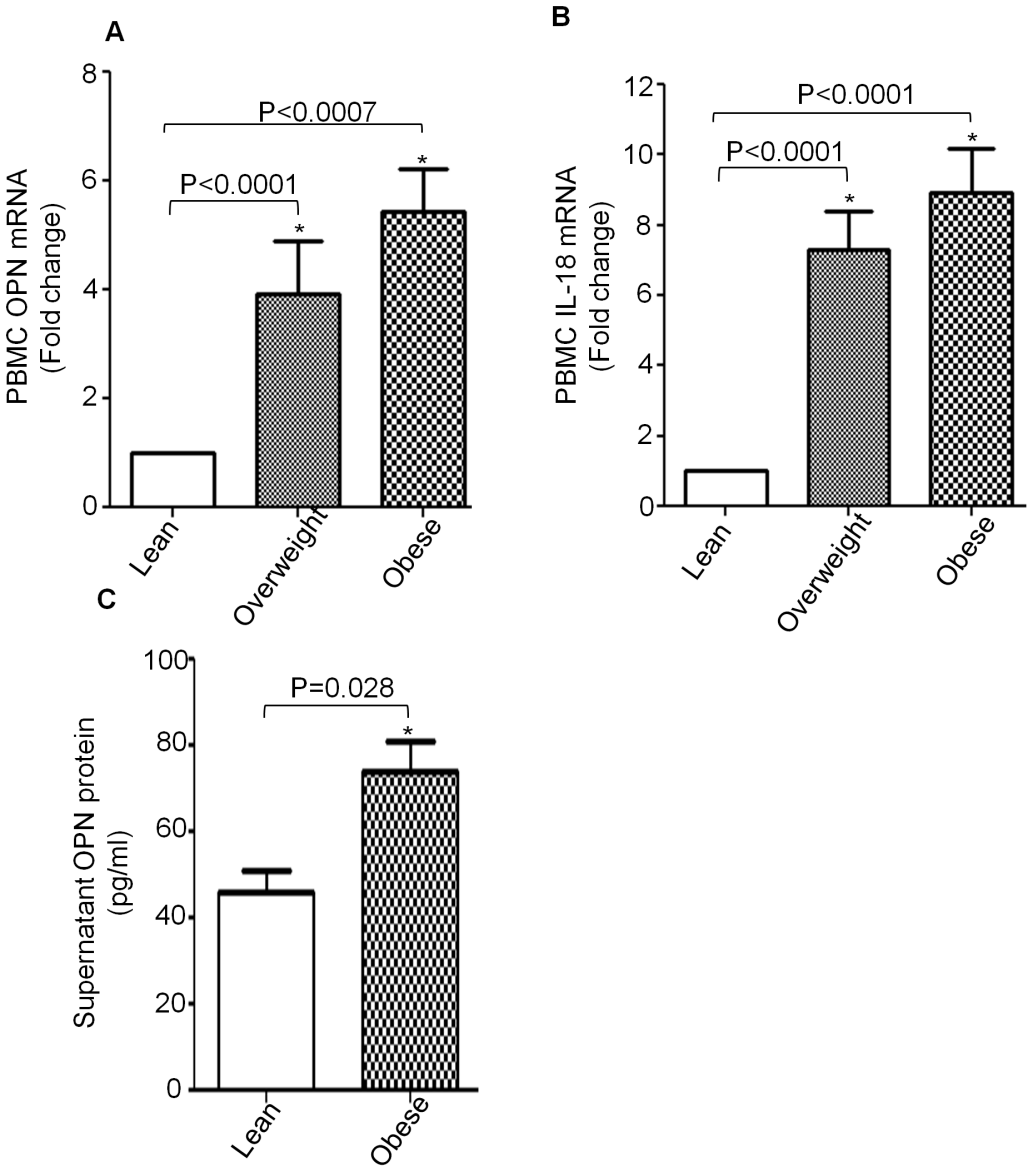
Expression of OPN and IL-18 by PBMCs. Total RNA was extracted from PBMCs of 60 individuals with different BMI (lean: 16; overweight: 20; obese 24). OPN and IL-18 mRNA were measured by real time quantitative PCR. Relative mRNA expression was expressed as fold expression over average of gene expression of lean group. The expression level in lean group was assumed to be 1. Values are presented as mean ± SE (A and B). PBMCs from three donors of each group were cultured in RPMI supplemented with 2% FBS for 16 hours and then culture supernatants were collected. Secreted OPN protein was determined in the culture supernatants by ELISA (C). Data presented were the means of three independent experiments performed with PBMCs of three donors of each group with ± SE.

As PBMCs are the main source for the production of both IL-18 and OPN, we next determined mRNA expression of OPN and IL-18 in PBMCs from lean (n: 16), overweight (n: 20) and obese (n: 24) individuals. mRNA expression of OPN and IL-18 was analyzed by quantitative real time RT-PCR. The level of OPN expression in the PBMCs from overweight and obese individuals was significantly higher (Obese: 5.4 Fold; Overweight: 3.9-Fold higher; P<0.05) than that in the control group (lean Individuals) (Fig. 2A). Similarly, the expression of IL-18 was dramatically upregulated in PBMCs from overweight and obese individuals (Overweight: 7.3 Fold; Obese: 8.9 Fold higher; P<0.05) than in the control group (Fig. 2B). Fig. 2C shows increased secretion of OPN by PBMCs from obese individuals compared to those from lean individuals (obese 73.96±6.78, lean 45.73±5.03 pg/ml; P = 0.028).

### Elevated Expression of OPN and IL-18 in Adipose Tissue

**Figure 3:**
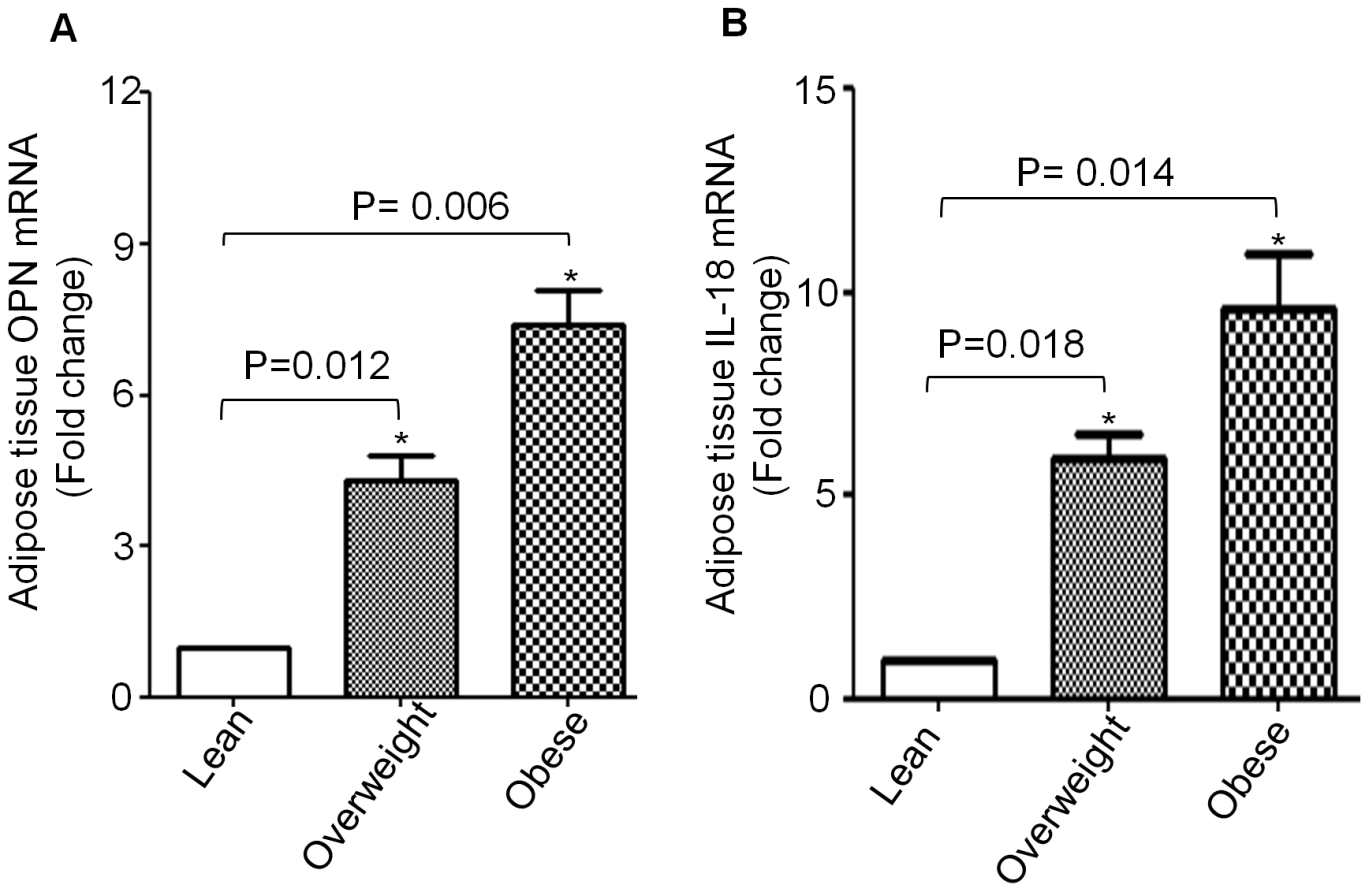
Expression of OPN and IL-18 mRNA in adipose tissue. Adipose tissue samples from 9 (lean: 3, overweight: 3 and obese: 3) individuals with different BMI were subjected to total RNA isolation. OPN and IL-18 mRNA expression was determined by real time quantitative RT-PCR. Relative mRNA expression was expressed as fold expression over average of gene expression of lean group. The expression level in lean group was assumed to be 1. Values are presented as mean ± SE (A and B).

Several studies suggest that adipose tissue is considered as an endocrine organ that produces and secretes a variety of cytokines, hormones and other metabolic players involved in the pathogenesis of obesity. Among this versatile group of mediators and effectors of inflammation, we have studied the expression of OPN and IL-18. All these markers, in their circulatory form, have been found. However, there is no much data available on their simultaneous expression in adipose tissue in human subjects. We isolated total RNA from subcutaneous fat biopsies from 9 individuals with different BMI values. We then measured RNA expression of OPN and IL-18 using quantitative real time RT- PCR. OPN and IL-18 were highly expressed in obese individuals (OPN: 7.3 Fold; IL-18: 9.6 Fold; P<0.05) as compared with those with normal BMI (Fig. 3A and B). Overall inflammatory activity, in addition to adipose tissue mass, provided information on a pathogenetic link between obesity and OPN and IL-18.

### Association between OPN and IL-18 in Obese Individuals

**Figure 4:**
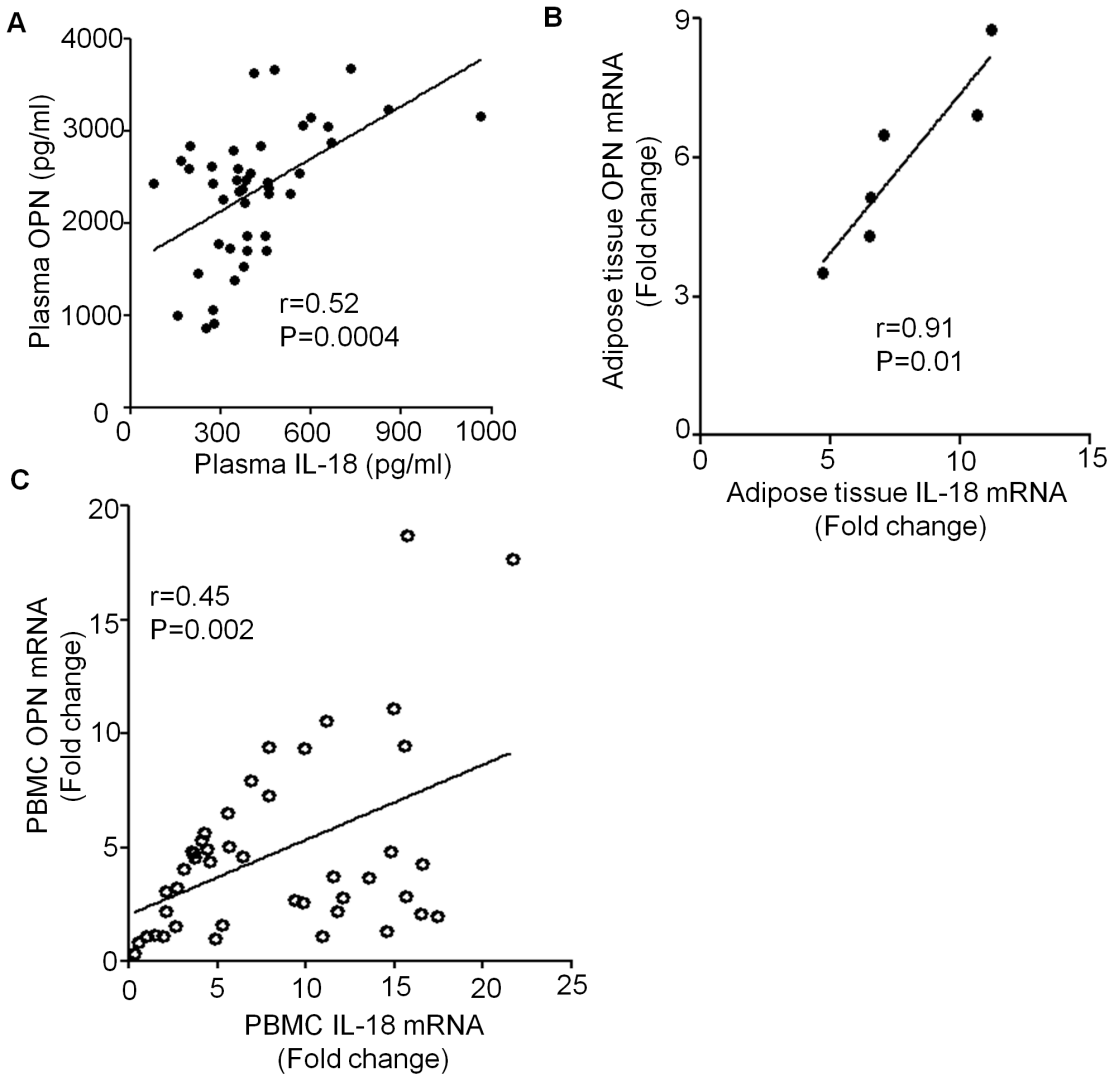
Linear regression between OPN and IL-18. Plasma OPN and IL-18 levels were determined by ELISA There was a statistical significant linear regression between OPN and IL-18 (r = 0.52, P = 0.0004, n = 44) in obese and overweight individuals which were free from overweight/obesity induced major complications (A). A strong association of OPN mRNA (Fold change) with IL-18 mRNA was observed in adipose tissue (r = 0.91, P = 0.01: (B). Fold change expression of OPN and IL-18 mRNA in PBMCs of 44 overweight and obese individuals was correlated with each other (r = 0.45, P = 0.0018) (C).

As our data show that OPN and IL-18 mRNA and protein levels were elevated in obese individuals, therefore, we investigated whether OPN had a relationship with IL-18 in obese individuals. To this end, we determined correlation between plasma OPN and IL-18 levels. As shown in Fig. 4A, we found a linear regression (r = 0.52, P = 0.0004) between IL-18 and OPN in obese individuals and overweight population. In addition, we observed a highly significant association (r = 0.91, P = 0.01) between OPN and IL-18 mRNA expression (Fold changes) in subcutaneous adipose tissue. (Fig. 4B). IL-18 gene expression (Fold changes) was significantly associated with OPN gene expression (Fold changes) in PBMCs from overweight and obese individuals (Fig. 4C; r = 0.45, P = 0.002). These results suggest that up-regulated IL-18 expression may contribute to increased OPN expression in obese individuals.

### OPN Expression was Induced in PBMCs by rhIL-18

**Figure 5:**
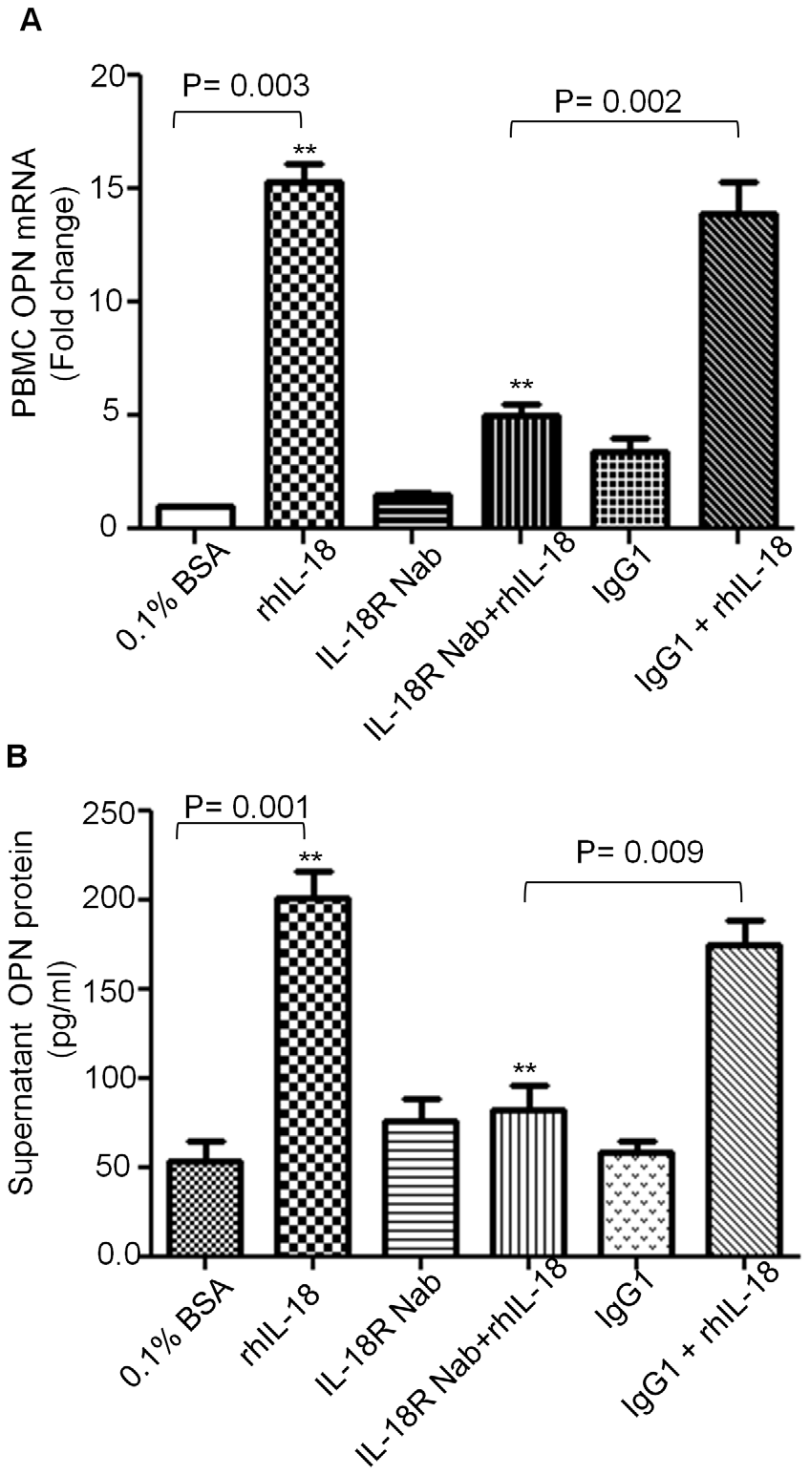
OPN is expressed by PBMCs and its secretion is induced by rhIL-18. PBMCs were treated with and without rhIL-18 (20 ng/ml) in the presence or absence of anti IL-18Rα neutralizing monoclonal antibody (0.1 ug/ml), and isotype control antibody IgG1 (0.1 ug/ml). Cells were treated as indicated for 16 hours. PBMCs and culture supernatants were harvested. Total RNA was isolated and OPN gene expression was determined by real time quantitative RT-PCR. Relative mRNA expression was expressed as fold expression over average of gene expression of controls. The average expression level in controls was assumed to be 1. Values are presented as mean ± SE (A). Secreted OPN protein was determined in the culture supernatants by ELISA (B). Data were shown from three independent experiments, each performed with PBMCs derived from three different healthy donors.

Since both OPN and IL-18 were found elevated in obese individuals, IL-18 could activate OPN expression in the PBMCs. To check this, PBMCs were cultured in presence or absence of rhIL-18. An increased expression of OPN, both at mRNA (Fig. 5A) and protein levels (Fig. 5B), was observed in PBMCs stimulated with rhIL-18 (P = 0.003). On the other hand, neutralization of IL-18Rα with an anti-IL-18Rα mAb led to the inhibition of IL-18-induced OPN gene expression (P = 0.002). In this case, OPN gene expression levels were similar to those observed in untreated cells whereas there was no change in OPN expression in cells treated with isotype control antibody.

### Association between Metabolic Parameters and OPN/IL-18

We further investigated whether the interaction between IL-18 and OPN had an effect on clinico-metabolic parameters. In this regard, serum levels of triglyceride, total cholesterol, high-density lipoprotein cholesterol, low-density lipoprotein cholesterol, lipoprotein, fasting blood glucose and HbA1C were also measured. FBG associated significantly with OPN (r = 0.32, P = 0.02) and IL-18 (r = 0.406, P = 0.0042) levels (Table 2). HDL, LDL, and cholesterol levels did not correlate with either IL-18 or OPN. However, BMI showed a strong correlation with IL-18 (r = 0.344, P = 0.016) and OPN (r = 0.499, P = 0.0002) levels. Moreover, FBG was significantly correlated with gene expression of OPN (r = 0.517, P<0.0001) and IL-18 (r = 0.259, P = 0.045) in PBMCs. Similarly HbA1C was associated with gene expression of OPN (r = 0.404, P = 0.0014) and IL-18 (r = 0.267, P = 0.043). Stepwise multiple linear regression analysis was performed to identify clinical variables (metabolic parameters) associated with OPN or IL-18 as a dependent variable. This analysis revealed that plasma OPN and IL-18 are independently associated with BMI (P<0.05) (Table 3). Similarly PBMC gene expression of OPN and IL-18 were also independently associated with BMI (P<0.05). Moreover, circulating FBG was a predictor of IL-18 (P = 0.004) and circulating TGL is a predictor of OPN gene expression in PBMCs (P<0.003) (Table 3).

**Table 2:** Correlation of plasma and PBMC gene expression levels of OPN and IL-18 with metabolic markers.

Inflammatory Mediator	Metabolic Parameters	Plasma		PBMCs	
		Pearson r	P	Pearson r	P
OPN	BMI	0.4995	0.0002***	0.5171	<0.0001***
	Fasting Blood Glucose (mmol/l)	0.3267	0.0206*	0.2644	0.0412*
	HDL cholesterol (mmole/l)	0.1220	0.3986	−0.2084	0.1070
	LDL cholesterol (mmol/l)	−0.08	0.5653	−0.0048	0.9705
	Triglycerides (mmol/l)	0.14	0.2989	0.4614	0.002**
	Cholesterol (mmol/l)	−0.1214	0.4012	0.2480	0.0539
	HbA1C	0.2550	0.0770	0.4041	0.0014**
IL-18	BMI	0.3443	0.0166*	0.4308	0.0003***
	Fasting Blood Glucose (mmol/l)	0.406	0.0042**	0.2596	0.0452*
	HDL cholesterol (mmole/l)	0.211	0.148	−0.2988	0.019*
	LDL cholesterol (mmol/l)	−0.2518	0.0877	−0.0106	0.9357
	Triglycerides (mmol/l)	0.2431	0.095	0.2472	0.0569
	Cholesterol (mmol/l)	−0.066	0.654	0.0187	0.8861
	HbA1C	0.300	0.0405*	0.2677	0.0426*

**Table 3:** Multiple stepwise linear regression analysis to identify clinical variables (metabolic parameters) associated with OPN or IL-18 level as a dependent variable.

Inflammatory mediators	Metabolic Parameters	Plasma		PBMC	
		β	P value	β	P value
OPN	BMI	0.516	<0.001	0.400	0.001
	Triglycerides	–	–	0.341	0.003
IL-18	BMI	0.283	0.034	0.376	0.003
	Fasting Glucose	0.388	0.004	–	–

## Discussion

Obesity induces chronic, low-grade tissue inflammation which is responsible, at least in part, for the development of obesity-related pathologies such as insulin resistance and type 2 diabetes, as well as cardiovascular diseases [1], [2], [3]; however, the cause and stimulus of persistent inflammatory activation in obesity largely remains unknown. It is speculated that migration of the inflammatory monocytes to the adipose tissue can be a major factor involved in obesity-induced inflammation. Both OPN and IL-18 are known as key mediators involved in different immunological disorders. Previous studies have shown that these two inflammatory mediators are abnormally elevated in obese individuals [32], [33], [34] It is noteworthy here that all the previous studies on OPN and IL-18 individually measured levels of these cytokines in different subject populations. To our knowledge, no study so far has shown that both inflammatory cytokines are simultaneously elevated in the same study population. Moreover, an interaction between OPN and IL-18 has not been elucidated. Here, our data show that plasma concentrations of OPN and IL-18 cytokines were simultaneously increased in obese individuals as compared with lean individuals and the levels correlated positively to each other. In addition, PBMC expression of OPN was high in obese individuals which also correlated with IL-18 expression by same cell population. It is previously known that OPN is highly up-regulated in adipose tissue from obese humans and mice, and is functionally involved in the pathogenesis of obesity-induced adipose tissue inflammation and associated insulin resistance in mice. Several studies in mouse models supported the role of OPN as a potential factor involved in obesity-induced complications [19], [35]. Circulating levels of IL-18 are elevated in obesity and correlate with body mass index, adiposity and insulin resistance [34]. Not surprisingly, IL-18 concentration is high in adipose tissue. In the present study, the data show that the expression of both OPN and IL-18 was simultaneously increased in the adipose tissues from overweight/obese individuals. These findings support a model in which adipose tissue OPN is progressively increased with the increase of IL-18 levels. Interestingly, as opposed to lean individuals, OPN expression in obese individuals was found to be elevated in the circulation as well as adipose tissue.

We argue that IL-18 levels are elevated during obesity-induced inflammation, a condition which favors upregulation of the OPN in circulation and adipose tissue. This prompted us to investigate whether OPN was expressed in response to IL-18 activation of PBMCs. In this regard, we show for the first time that OPN expression was induced in PBMCs treated with rhIL-18. Similarly, a murine model study demonstrated parallel increases in IL-18, OPN expression and interstitial fibrosis in primary cardiac fibroblast cultures [36]. IL-18 activates the transcriptional factors AP-1 and NF-κB in T lymphocytes that show upregulated transcription of the OPN gene [37], [38]. We also observed that the *ex vivo* production of OPN by cultured PBMCs was higher in obese than lean individuals. This may be due, in part, to the fact that t cells and monocytes/macrophages in obese individuals, unlike their lean counterparts, are in a state of chronic activation which, further leading to enhanced production of OPN by these immune regulatory cells. Increased circulatory levels of OPN will further exacerbate immune cell activation. Apart from Th1-cell activation, OPN can also activate macrophage adhesion, migration, and cytokine release [15], [39]. Our present findings, therefore, support the increased *ex vivo* production of OPN during the process of obesity induced inflammation.

Chronic systemic inflammation is considered to play an important role in the development of diabetes and it may represent a unifying link between metabolic syndrome, type 2 diabetes, and cardiovascular disease. To explain a link between inflammation and insulin resistance, it was proposed that OPN might cause accumulation of the macrophages in adipose tissue and lead to an increased macrophage-related inflammatory activity [19], [40], [41]. Consistent with the previous cross-sectional studies [26], [42], [43], our data also show that the circulating IL-18 and OPN levels increased progressively with the escalating BMI values. After stepwise multiple linear regression analysis both OPN and IL-18 were found to be independently associated with BMI (P<0.05) which indicated that obesity contributed to the upregulation of OPN and IL-18 levels in plasma and in PBMCs. This independent association of OPN and IL-18 with obesity may aggravate inflammation status in obese individuals and affect insulin resistance as well. Although a significant correlation of OPN and IL-18 with FBG, multiple regression analysis detected only significant independent association of IL-18 with FBG, indicating that the relationship of IL-18 with FBG is dependent on obesity. Since obesity has independent association with both OPN and IL-18, OPN and IL-18 showed a positive correlation with the fasting blood glucose which may be taken as a reasonable surrogate measure of the insulin resistance. Previously it has been shown in different studies that high expression of OPN and IL-18 correlated with obesity-induced inflammation and insulin resistance. [32], [33], [34], [44], [45]. In this particular scenario, the relationship between OPN and IL-18 could be very crucial in development of insulin resistance in obese individuals.

In summary, we show for the first time that plasma, PBMCs and adipose tissue OPN and IL-18 levels are simultaneously increased and correlate with each other in overweight/obese individuals which may trigger the development of insulin resistance. This study also provides the direct evidence that IL-18 regulates OPN production in the PBMCs. In view of the above results, this interaction between two proinflammatory mediators may serve as a potential disease marker for monitoring insulin resistance severity and therapeutic efficacy. Importantly, obese individuals with a combined OPN/IL-18 elevation may be at a greater risk for developing insulin resistance as compared with non-obese population.
